# Modeling and Design Optimization of a New Piezoelectric Inchworm Actuator with Screw Clamping Mechanisms

**DOI:** 10.3390/mi13122038

**Published:** 2022-11-22

**Authors:** Haichao Sun, Yunlai Shi, Qiang Wang, Xing Li, Junhan Wang

**Affiliations:** 1 State Key Laboratory of Mechanics and Control of Mechanical Structure, Nanjing University of Aeronautics and Astronautics, Nanjing 210016, China; 2 School of Electrical and Mechanical Engineering, Pingdingshan University, Pingdingshan 467000, China

**Keywords:** piezoelectric actuator, dynamic model, numerical method, design optimization

## Abstract

A new piezoelectric inchworm actuator with screw clamping mechanisms has been developed recently for the wing folding mechanism of a small unmanned aircraft where the actuator power density is a great concern. Considering that the prototype actuator was designed just with engineering intuition and the performance optimization through experimental developments would take a vast amount of cost and time, a mathematical model was developed to investigate the actuator’s critical design parameters and optimize its presently undesirable performance. Based on the lumped parameter method reported previously, and taking full account of the detailed modeling of the complex actuator housing and the actual nonlinear behaviors from the high-force contact and friction occurring at the screw-nut interface, as well as the output performance of the main drive elements including the piezoelectric stack and hollow ultrasonic motors (HUSMs), this model was built and then was experimentally verified for its accuracy and availability. Finally, nine design parameters were studied for their individual effect on the actuator’s output using the proposed model. The simulation results indicate that the performance can be considerably improved by performing a slight modification to the prototype, and the dynamic modeling and parameter optimization methods used in this study can also serve as a useful reference for the design of similar piezoelectric inchworm actuators with intermittent clamping behaviors.

## 1. Introduction

**Figure 1:**
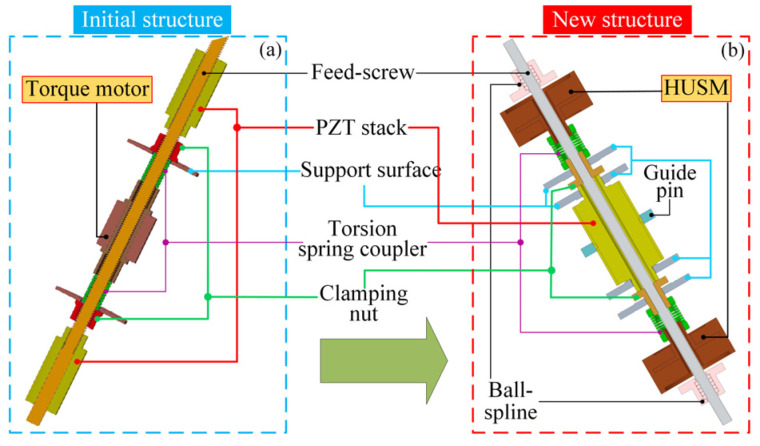
Improvement of design. (**a**) Initial structure; (**b**) new structure.

Many piezoelectric actuators (PAs) with a long stroke and a high force output have been developed for various smart structural applications, benefiting from the piezoceramics’ attractive merits such as a high energy density, rapid response, high positioning precision, flexible shape, and so on [1,2,3]. With consistent evolutions in design and steady improvements in performance, PAs are approaching a level comparable to that of traditional electromagnetic motors and hydraulic actuators [4,5,6]. In general, motion amplification and rectification are essential in most PAs, for example, ultrasonic motors (USM) rectify a tangent part of the trajectory motion of the particles lying on the surface of the resonant stator excited by piezoceramics to realize a linear or rotary motion; inertial actuators accumulate the slide portion of the stick-slip motion between the mover and stator—usually produced by a piezoceramic-driven flexible motion amplification mechanism—to achieve a mechanical power output; and piezoelectric inchworm actuators (PIAs) often utilize displacement-amplified clamping devices to rectify the micro electric-induced strains caused by a PZT (which is an abbreviation for the most common piezoceramic material, Pb (ZrTiO3)) stack-driven element [7,8]. The characteristics of the three kinds of PAs described above have been demonstrated clearly in plenty of previously published literature, and comparatively speaking, PIAs display a great potential in terms of the load capacity and high output power [1,9]. Noticeably, Loverish et al. from Penn State University proposed a passive screw–nut clamping design with a self-locking function instead of an active friction clamping mechanism in the development of a novel PIA [10]. The high load capacity and favorable prospects in the wind folding applications of an unmanned aircraft, demonstrated by the actuator and the robust stiffness and insensitivity to wear given by the helical clamping mechanisms, drew our attention greatly. Then, Shi et al. in our research group employed two symmetrically configured PZT stacks to improve Loverish’s prototype and solved the issue that the actuator is only capable of pushing against a compressive load and it is unable to actuate the tensile load in the reverse direction because of the anti-compression and poor pulling properties of the PZT stack, as shown in Figure 1a [11]. Regrettably, the longer size envelope employed to accommodate an additional PZT stack, as well as the undesirable mechanical performance in the output force and power density (i.e., the power to weight ratio), is probably due to the high degree of structural compliance and the fact that only a single PZT stack is activated to drive a load in a unidirectional operation while the other one remains dormant, which seem to be major obstacles for widespread applications. Recently, an improved design, following a preceding feed-screw actuator study conducted by our research team, was put forward and developed for a morphing aircraft project sponsored by the National natural science fund, as shown in Figure 1b. The significant discrepancies between these two designs could be summarized in two points: first, only one PZT stack was utilized in the middle of two clamping units to withstand the compressive or tensile load during the actuator’s operation, in order to remove redundancy and compact the structure; second, instead of the torque motor, two hollow USMs were located on either side of the PZT stack, driving the clamping nuts independently to relieve the kinematic coupling between the piezoelectric element and the clamping unit. The experimental results indicate that the current prototype, using only a single PZT stack scheme instead of the original dual-PZT stack configuration, is not only able to achieve a higher power density and a more compact structure but is also able to produce a rated load of approximately 500 N (corresponding to its peak output power) and a blocked force of 700 N, each of which significantly exceeds double the value of the former design. Although the progress is impressive, the development strategy based only on engineering intuition and the performance gap between the prototype actuator and actual application requirements are still necessary to address. Considering that the implementation of dozens of experimental investigations and tests in order to perfect the hardware would present a daunting challenge, a dynamic mathematical model describing the actuator’s behaviors was developed and used for a series of design optimization research in this study.

Essentially, the proposed PIA at work is a vibratory system, in addition to the stiffness and inertia of every component, the discontinuous interface properties, including the normal contact and tangential friction occurring between the feed screw and clamping nuts, and the nonlinear mechanical characteristics of all the drive parts, such as the PZT stack, and USMs more significantly affect the modeling accuracy and solution efficiency, and of course, become important things to deal with. It is worth noting that some available numerical modeling methods for this kind of motion-accumulated actuator reported previously provide valuable references for us. First of all, Mockensturm et al. proposed a lumped parameter model in their roller-wedge piezoelectric actuator studies. The simulated results fit well with the experimental results in addition to underestimating the output power by an average of 26.4% near stall torque [12]. Then, Loverich et al. applied this numerical method to model the screw-clamping-type PIA mentioned above [13]. Although the model captured the overall variation trend of the actuator’s behavior, the simulation fidelity was poor at the low and high load stages, as the authors described, and an average deviation of 36.1% and 10.1% relative to the experimental values in speed could be estimated, respectively, for both load stages according to the published simulation and experiment results, probably because, in their formulation, the sub-models, such as the PZT stack, torque motor, and normal contact model for the helical clamping unit, were simplified and linearized. Additionally, the actuator’s housing cross-section was simply taken to be identical along its length on the basis of engineering experiences for acquiring a high computation efficiency. Recently, a few inertial actuator models based on the lumped parameter method have been published, but modeling research on inchworm actuators is seldom seen [14,15]. This study firstly built on the previously published modeling and optimization techniques by proposing a more effective, more accurate dynamic mathematical model for the new prototype actuator, where the real performance nonlinearity of all the actuation components, such as the PZT stack and hollow USMs and the structural variations between the new and the old designs, especially between both quite-different actuator housings, were synthetically considered, and the revision-by-experiment method was adopted duly, then a system of parameter studies to optimize the performance of the actuator using the refined dynamic model was conducted. The remaining part of this paper is organized as follows: Section 2 presents the actuator’s structure and performance, then the detailed modeling process is described in Section 3. Afterward, Section 4 gives the experimental validation of the model, sequentially, and the parameter investigation and optimization analysis are discussed in Section 5. Finally, we conclude this study in Section 6.

## 2. Descriptions of the Actuator

The research object in this paper is an improved design from our previous work, as shown in Figure 1b. The newly developed linear piezoelectric actuator was also designed as a symmetric configuration, which principally consisted of a tubular PZT stack sandwiched between two helical clamping units. In each helical clamping unit, a clamping nut is driven by a hollow ultrasonic motor (HUSM) via a torsion spring coupler while it mates with the feed screw to achieve the clamping function when contacting the corresponding support surface. The feed screw (which can also be regarded as the mover of the actuator) is assembled through the middle hole of each component, and two ball splines are installed on both ends of the actuator to limit the rotation of the feed screw. The PZT stack is restricted in rotary degrees of freedom by several symmetrically arranged guide pins. Four support surfaces are configured to accommodate and hold the PZT stack or the clamping nuts. Pins, ball splines, HUSMs, and support surfaces are attached firmly to a rigid actuator housing. The notable differences between this new scheme and the old one have been introduced in the Introduction.

**Figure 2:**
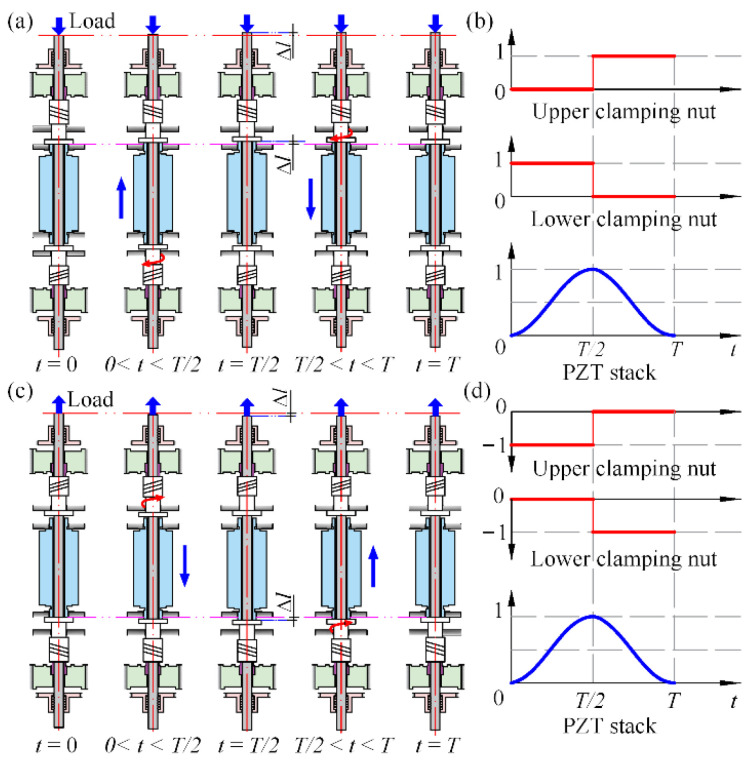
Operation principle of the actuator. (**a**) Operation in forward motion, (**b**) motion state of the components in forward motion, (**c**) operation in backward motion, (**d**) motion state of the components in backward motion.

Similar to the operation principle of a common PIA, the working process of the proposed actuator involves an alternate rotation of two nuts on a feed screw to periodically accumulate the PZT stack’s micro displacement, as presented in Figure 2. The working states of the PZT stack and clamping nuts are illustrated in Figure 2b,d. For the clamping nut: ‘1’ means ‘the clockwise rotary direction; ‘−1’ donates ‘the counter-clockwise rotary direction; and ‘0’ represents ‘stop’. For the PZT stack: ‘1’ indicates ‘the maximum extension state’ while ‘0’ signifies ‘the original state’. The detailed operating procedures for actuating a pressure load (see Figure 2a,b) are described as follows:

1. At the initial state, when the appropriate drive signals have been applied, the HUSMs rotate the upper and the lower clamping nut around the feed screw (without translating) downward onto the top of the PZT stack and the bottom support surface, respectively, and the PZT stack is further pushed onto the lower support surface above the bottom one.
2. From time 0 to *T*/2, with the supplied periodic voltage excitation, the stack extends and advances the upper clamping nut together with the loaded feed screw upwards, while the lower clamping nut is screwed along the feed screw by the lower HUSM downward to the bottom support surface. In this process, the rotation of the upper clamping nut following the upper HUSM is restrained due to the large friction force between the nut and PZT stack, while that of the lower clamping nut is free due to the separation between the nut and the bottom support surface.
3. When at *T*/2, if the operations are coordinated, the PZT stack reaches its maximum stroke (Δ*l*) while the lower clamping nut is rotated down to the bottom support surface, thus the loaded feed screw can be held by that support surface when the PZT stack recoils during the next half of the stack driving period.
4. During the time from *T*/2 to *T*, the PZT stack relaxes, and the upper clamping nut is rotated down by the upper HUSM together with the stored rotational potential energy of the upper torsion spring and tracks the PZT stack contraction. Meanwhile, the lower clamping nut stops rotating because it is tightly pressed on the bottom support surface.
5. At time *T*, the PZT stack returns to its original length and the upper clamping nut has been screwed onto the top of the stack again.

Thus, the actuator completes one periodical actuation wherein the feed screw moves forward one step. Repeating the steps from two to five can produce a continuous power output, and the travel of the actuator depends on the length of the feed screw theoretically, which is flexible and increasable.

As for the working process for powering a tensile load in the reverse direction for the linear actuator, it is similar to that described above for driving a compressive load because of the structural symmetry; the only differences are that the PZT stack is pressed onto the corresponding upper support surface, and the two clamping nuts’ roles and rotary directions are just reverse to that operated for actuating a compressive load, which is displayed in Figure 2c,d.

**Figure 3:**
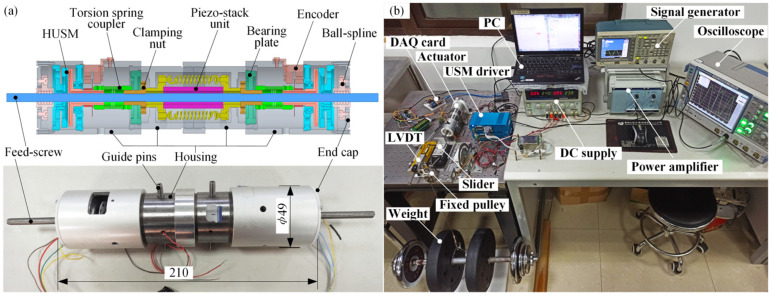
Prototype and experimental setup. (**a**) Prototype; (**b**) setup.

According to the above design and analysis, a CAD model was constructed, and a proof-of-concept prototype which was 210 mm in length, 49 mm in outer diameter, and 0.82 kg in weight was manufactured and tested by recording its average speed for a discrete range of suspended weights, as pictured in Figure 3. The experiments reveal that the actuator not only demonstrates a higher power density and a more compact structure than our previous development but that it also produces an about 500 N rated load and a 700 N blocked force, any of which significantly exceeds twice the value of the original one. Although it performs well among most piezoelectric actuators, the 1.34 W/kg power density and the stall force, less than one-fourth of the stack blocked force (≈3300 N estimated by empirical formula), of the prototype are unsatisfactory compared with that of the traditional electromagnetic actuators with a similar power output. Therefore, a mathematical model was constructed below to further exploit the possibilities of the linear actuator concept.

## 3. Modeling of the Actuator

**Figure 4:**
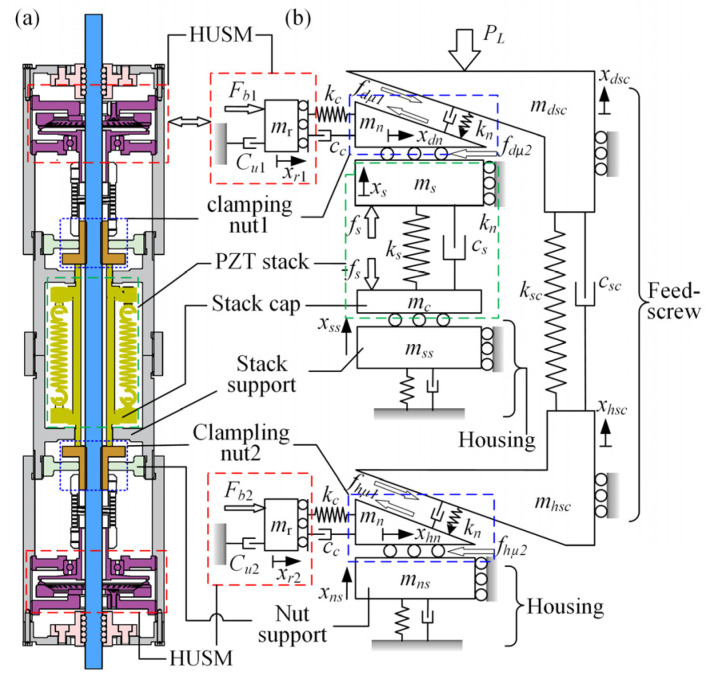
The actuator model. (**a**) CAD model; (**b**) dynamic model.

A large number of dynamic models for piezoelectric actuators have been reported during the past few decades, and the methods could be summarized in two types: the analytical method and the numerical one [16,17,18]. Generally, the former is suitable for a simple and regular structure such as the USM, while the latter is good at simulating a discrete and complex system. Clearly, the discontinuous interface behavior occurring in the screw–nut clamping mechanisms and the discrete distribution of every component render the lumped parameter method more conducive to describing the prototype. The central idea of this numerical modeling is that systems are simplified by approximating continuous elastic components as discrete or finite lumped parameters, including the mass, linear spring, and viscous damper, so that they can be characterized by a set of ordinary differential equations [12]. It should be noted that the model accuracy is usually proportional to the number of the applied degrees of freedom, which conflicts with the solution efficiency; then, hence, founding on the predecessors’ study while balancing the precision and time cost, the actuator model was constructed and plotted in Figure 4. The model mainly consists of the following four sub-models.

### 3.1. The Stack Model

**Figure 5:**
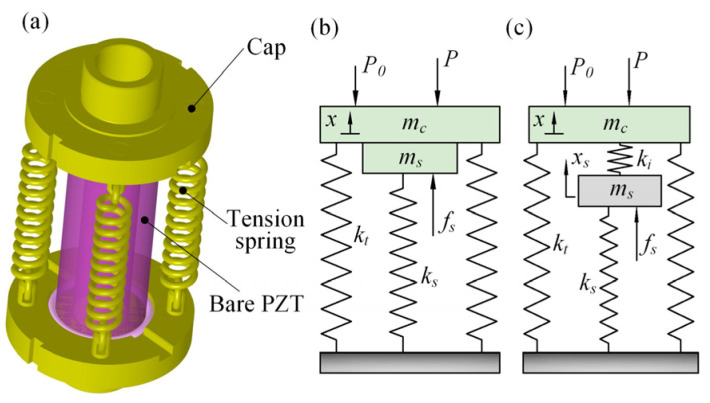
Analysis of PZT stack. (**a**) Structural layout; (**b**) ideal equivalent model; (**c**) new equivalent model.

**Figure 6:**
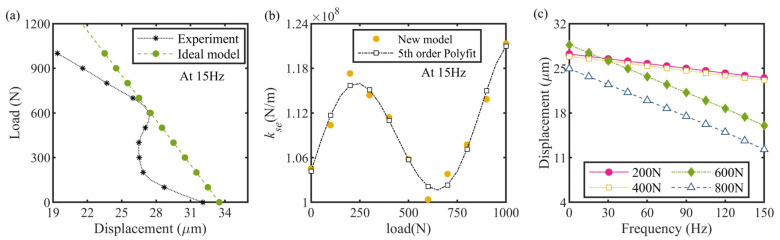
PZT stack’s performance. (**a**) Load versus displacement; (**b**) equivalent stiffness versus load; (**c**) displacement versus drive frequency.

Usually, a bare PZT stack should be placed into an elastic preload mechanism (a tension-spring preload mechanism here) in order to avoid damage and realize an excellent dynamic characteristic, as presented in Figure 5a. Based on the piezoelectric constitutive equations, bare PZT stacks could be approximated by a single-degree-of-freedom body with a stiff spring (*k*_s_), end-mass (*m*_s_), and exciting force (*f*_s_), where the end-mass was estimated to be one-third of its total mass, assuming the density is uniform along the length, and the external force *f*_s_ was determined by the product of the blocked force *F*_b_ and the ratio of the stack drive voltage *u*_i_ to its maximum operating voltage *U*, namely *f*_s_ = *u*_i_*F*_b_/*U*. Thus, the PZT stack could be modeled as a spring–mass system with an initial preload *P*_0_ ideally, as shown in Figure 5b. Ignoring the damping and inertia due to its low operating frequencies, the stack’s external load (*P*) as a function of the displacement (*x*) could be given by:

(1) $$P=f_{s}-P_{0}+\left(k_{s}+k_{t}\right)x$$

where *k*_t_ is the stiffness of the tensile springs. Unexpectedly, the load versus the output displacement curve measured experimentally at a frequency of 15 Hz for the PZT stack is not coincident with that predicted by Equation (1) (see Figure 6a), which likely results from the neglected stiffness of bond lines between the bare stack and cap even among the piezoceramics, whereby a new stack model integrating the internal bond layers’ stiffness (*k*_i_) was constructed and shown in Figure 5c, again giving the expression of the external load *P*:

(2) $$P=f_{s}-P_{0}+\left(k_{s}+k_{t}+k_{s}k_{t}/k_{i}\right)x$$

Notably, if we set *f*_se_ and *k*_se_ equal to the constant item, (*f*_s_ − *P*_0_), and the first order item, (*k*_s_ + *k*_i_ + *k*_s_*k*_t_/*k*_i_), of Equation (2), respectively, the two parameters could also be regarded as the exciting force and equivalent stiffness of the PZT stack model in sequence. Assuming that the internal stiffness *k*_i_ varies with the load *P* during the stack’s motion, the dependence of the equivalent stiffness *k*_se_ on the load trend was computed and plotted in Figure 6b, according to Equation (2) and the experimental data presented in Figure 6a, and simply a fifth order polynomial gave a desirable matching. In addition, the linear relationship between the tested displacement and drive frequency shown in Figure 6c was also introduced into the PZT stack model.

### 3.2. Model of the Clamping Mechanism

The screw–nut clamping unit could be usually simulated by a plane wedge block, as shown in Figure 4, where the wedge angle was equal to the screw pitch angle and the nut mass was calculated by the ratio of its inertia to the square of its radius. For simplicity, the equivalent stiffness for a homogeneous bar or tube-shaped structure, such as the feed screw and actuator’s housing discussed below, was reckoned by a ratio of the product of its material’s Young modulus and sectional area to the effective length while the structural damping for the actuator’s components was determined based on their material loss factors. In addition, as a significant part influencing the model’s accuracy and time cost directly, the screw–nut interface model that characterizes the discontinuous high-force contact and friction behavior should be treated with discretion here. The most convenient approach for modeling the contact of the rough surfaces is the probabilistic method, which begins with the study of a single asperity’s behavior on a microscopic scale and then incorporates it in a statistical model of multiple asperity contact [19]. There have been many typical asperity contact models considering the different deformation processes published so far, such as the Hertz, CEB, ZMC, KE, JG model, and so on [20,21,22,23]. However, the purely elastic Hertz model proposed by Greenwood seems suitable for long-term intermittent contact in this study, whereupon the contact load is given:

(3) $$P_{e}=4E\times R^{\frac{1}{2}}W^{\frac{3}{2}}/3$$

and

(4) $$\left\{\begin{array}{l} E^{*}=(E_{2}\left(1-\upsilon_{1}^{2}\right)+E_{1}\left(1-\upsilon_{2}^{2}\right))/\left(E_{1}E_{2}\right)\\ R=R_{1}R_{2}/\left(R_{1}+R_{2}\right) \end{array}\right.$$

where *E*_1_ and *E*_2_ are the Young’s moduli, *ν*_1_ and *ν*_2_ are the Poisson’s ratio, and *R*_1_ and *R*_2_ are the radius of the two contacting asperities by turns.

**Figure 7:**
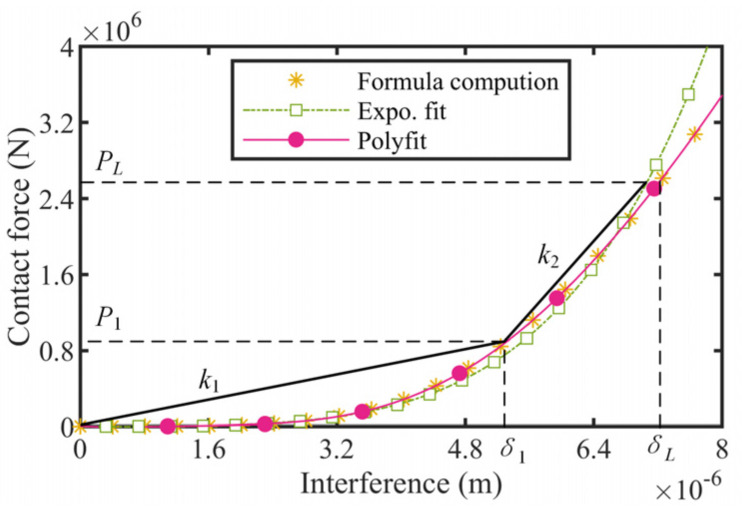
Approximation of screw–nut contact characteristic.

Using the common Gaussian function *ϕ*(*y*) (refers to Equation (5) in the surface interface model to describe the distribution probability of the asperity heights (*y*)), the contact load between the two contacting surfaces can be derived by a definite integral over a specified range of the actual distance between both the contacting surfaces. *d*:

(5) $$\phi \left(y\right)=e^{-y^{2}/\left(2\sigma^{2}\right)}/\left(\sqrt{2\pi }\sigma \right)$$

(6) $$P_{n}=\eta A_{n}\int_{d}^{+\propto }P_{e}\phi \left(y\right)dy$$

where *η* and *A*_n_ are the density of the asperities and the nominal contact area, respectively. *δ* = *y* − *d* is defined as the interference usually. Due to the similar material composition to the used nut and feed screw, a set of surface topographical parameters measured by Nuri and Haling (1975) was adopted here [24]. Via a numerical integration of the above equations, the contact force versus the interference trend for the screw–nut pair was solved theoretically and plotted in Figure 7. Obviously, the contact force goes up nonlinearly with the rise in the interference, and so does the contact stiffness. Realizing that directly introducing this nonlinearity to the model would increase the solution difficulty considerably, we took a critical load *P*_1_ as a breakpoint and separated the varying process of the contact force into two distinct linear phases to capture both crucial scenarios occurring in the discontinuous clamping motion (see Figure 7): (1) the maximum screw–nut load *P*_L_ and (2) the load *P*_1_ at which the HUSM begins to rotate the nut (which could be derived easily from a force analysis performed for the helical clamping model), because the first load point dominates the precise reduction in the PZT stack’s stroke that is transferred to the load due to the structural compliance of the actuator, while the other point determines the start and stop conditions of the displacement-accumulated motion and dramatically influences the nut’s dynamics at high frequencies, thus, giving a bi-linear stiffness approximation for the nut-screw contact process. Additionally, this treatment had been verified well previously by the aforementioned Loverish research team. In addition, because of the discrepancy between the theoretical and actual surface contacting property, the rigidization treatment of several contact surfaces such as the PZT stack-support surface pair and the stack cap-nut pair, we brought an adjustment coefficient, *k*_a_, in the contact model to reconcile the actuator model’s prediction and experimental results. Thereby, the nonlinear trend of the contact force relative to the interference shown in Figure 7 can be fitted by the following polynomial:

(7) $$P_{n}=k_{a}\left(5.2e^{37}\delta^{6}-1.5e^{33}\delta^{5}+1.4e^{28}\delta^{4}+5.0e^{22}\delta^{3}+8.2e^{16}\delta^{2}-5.0e^{10}\delta +7050\right)$$

where *P*_n_ and *δ* represent the contact force and interference, respectively. Substituting *P*_L_ and *P*_1_ to Equation (7) yields *δ*_L_ and *δ*_l_, then the linear contact stiffness *k*_1_ and *k*_2_ could be calculated.

Likewise, numerous surface friction models such as the speed-dependent model, LuGre model, MFR model, and so on have been proposed up to now, but the notable LuGre model was chosen for approximating the screw–nut friction behavior due to its simplicity, high efficiency, and convenience [25,26,27]. In such a model, the friction phenomenon was taken as the interaction of tiny bristles growing on contacting surfaces, and the Stribeck effect, hysteresis, and spring-like characteristic observed experimentally were captured and expressed by:

(8) $$\frac{dz}{dt}=v-\left|v\right|\frac{\sigma_{0}z}{h\left(v\right)}$$

(9) $$h\left(v\right)=f_{c}+\left(f_{s}-f_{c}\right)\exp\left[-{\left(v/v_{s}\right)}^{2}\right]$$

(10) $$f_{f}=\sigma_{0}z+\sigma_{1}\frac{dz}{dt}+\sigma_{2}v$$

where the mean deflection of the surface bristles was defined as the state variable *z*; *v* and *v*_s_ represented the relative and the stribeck speed, respectively; and *σ*_0_, *σ*_1,_ and *σ*_2_ were the bristle stiffness, damping coefficient, and viscous friction coefficient in the sequence, while *f*_s_ and *f*_c_ were individually the stiction and coulomb friction force. Occasionally, a simple conversion from the LuGre model proposed by Gaul and Nitsche in their study on the damping characteristic of a screw-thread joint was applied for this study due to the similar structure, and the reported experimental parameter values were borrowed [28].

### 3.3. The HUSM Model

**Figure 8:**
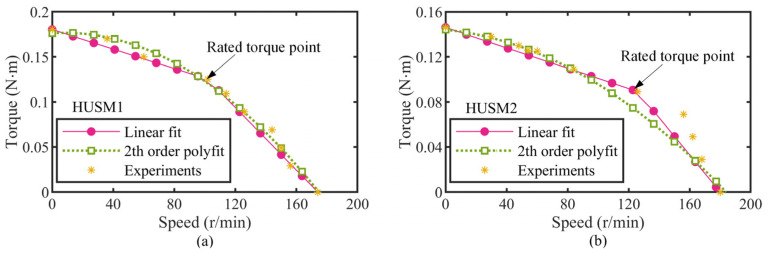
Performance of two HUSMs. (**a**) HUSM1; (**b**) HUSM2.

As demonstrated in Section 2, the output performance of the actuator depends on the coordination between the PZT stack dive frequency and the HUSM rotary speed. The larger the HUSM speed is, the higher the stack driving frequency able to be matched is, and assuming the extending stroke of the stack is constant at a fixed load, the actuator’s speed (*v*) can be defined as the product of the available stack stroke (*δ*) and its drive frequency (*f*) theoretically, which implies that the motor speed determines that of the actuator. Therefore, an accurate HUSM model is essential for the actuator’s modeling. Figure 8 gives the torque speed experimental results and the mathematical fitting results of the two HUSMs. Clearly, the bi-linear model determined by the stall torque, rated torque, and free speed point was preferable for describing the HUSMs’ performance.

### 3.4. The Housing Model

**Figure 9:**
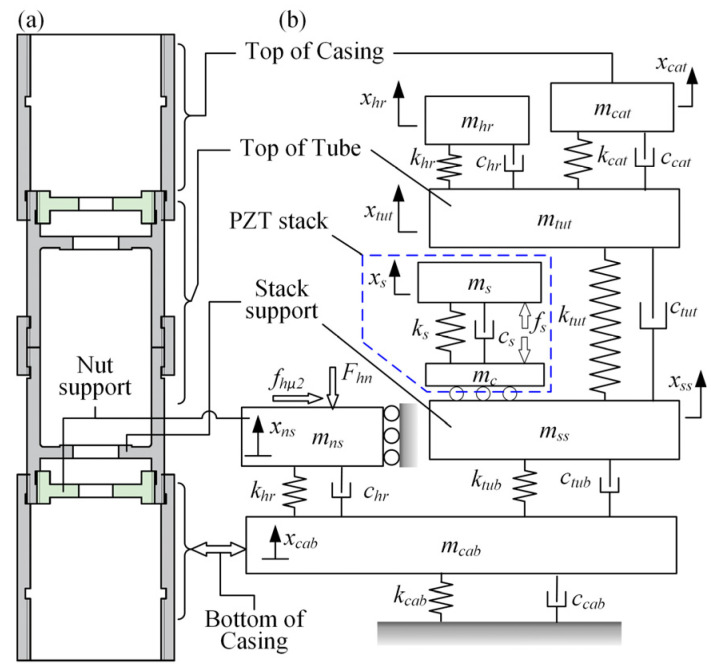
Actuator housing model. (**a**) CAD model; (**b**) equivalent model.

As shown in Figure 9a, the actuator’s housing mainly consists of casings and tubes, which are screwed together symmetrically and furnish accommodation and support for the driving and clamping units. Because its compliance substantially affects the really accumulated displacement in the PZT stack’s stroke, *δ*_r_, it should be taken carefully. Over a sufficient mechanical analysis and refinement, a detailed lumped parameter model for the housing was established in Figure 9b, in which the parameter values, such as the equivalent stiffness and viscous damping, were derived from the same methods as that mentioned in the feed screw model.

## 4. Model Validation

**Figure 10:**
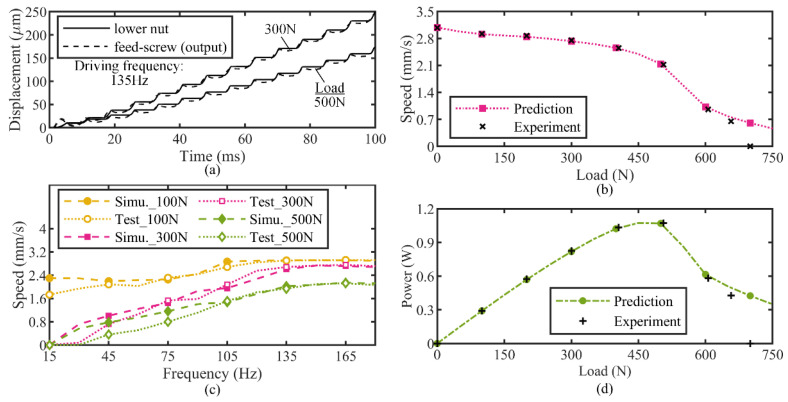
The output performance of the actuator. (**a**) Transient displacement of lower nut and feed screw; (**b**) speed versus load curve; (**c**) frequency-dependent characteristic; (**d**) power versus load plot.

According to the above analysis and modeling, a set of ordinary differential equations that govern the prototype’s response were established and verified according to Newton mechanics and Lagrange mechanics, respectively. Because of the intrinsic discontinuous characteristic occurring in the clamping motion, finding a high-efficiency and closed-form analytical solution for the system’s governing equations is difficult, and thus the numerical solution was pursued. There are lots of tools and methods for numerical solutions, in which the MATLAB Simulink platform, utilizing a series of diagram blocks with a specific function and signal-flowing arrows to describe the differential equations of a system and an explicit Runge–Kutta variable-step solver to perform the solutions, is an easy-to-use tool for simulating the actuator’s behavior. Similar to the actual experimental procedures, the output speed as a function of the stack drive frequency over a discrete range of a constant load was computed in Simulink, and the output displacement of the lower nut and feed screw versus the time traces at a 135 Hz drive frequency and the simulated output speed versus the frequency plots for several constant loads was given in Figure 10a,c, respectively, wherein Figure 10a reveals a minor back-driven (backlash) phenomenon occurring in every stepping drive period of the actuator due to the structural compliance, while Figure 10c shows a good agreement between the simulated results and the experimental data collected from the prototype at three levels of the external load. The slight divergence at frequencies below 75 Hz may result from the ignorance of the different torsional stiffness of two spring couplers working at the reverse rotary direction and their ignored angular displacement limit, but this is not of much concern as the actuator is always operating at a higher frequency for an optimal speed output.

Taking the peak speed at each constant load shown in Figure 10c as the actuator’s output speed, the actuator’s output power can be determined by the product of the output speed and the applied load. Figure 10b and d show the predicted and experimental actuator’s output performance results. A good match is also attained in the shape of both the speed versus load curve and the power versus load curve below the 650 N external load, and the agreement between the simulated and experimental results is apparently preferable to that of the congeneric inchworm actuator proposed previously by Loverich et al. within the low load range, according to Figure 10b and the descriptions in the Introduction. Near the stall load, the model overestimates the actuator’s performance output, which is likely due to the modeling tolerance between the ideally linearized treatment and the actual nonlinear characteristic for the PZT stack’s stroke-frequency performance in a heavy load range and the neglected inconsistent coupler stiffness. Nevertheless, the dynamic model predicted accurately the peak power point at an approximately 500 N load (a common concern in a motor development) and captured the overall varying trend of the prototype actuator’s motion, and consequently has the qualification for the subsequent parameter studies and optimization design of the device.

## 5. Parameter Studies and Design Optimization

Confident that the mathematical model could accurately predict the prototype actuator’s performance, the parameter studies and optimization analysis were conducted to investigate how the performance was improved further. Similar to a common vibration system, the stiffness and inertia of some critical components may greatly influence the actuator’s output response; also, the contradictory interaction between these two physical parameters usually makes the initial prototype design that only depends on engineering experiences un-perfect. Considering such characteristics and the actuator’s operation characteristic discussed above synthetically, nine crucial design parameters were studied for their individual effect on the actuator’s performance, and they were: (1) the wire diameter of the torsion spring coupler; (2) the housing thickness; (3) the thickness of the nut support; (4) the tube material in the housing; (5) the clamping nut diameter; (6) the feed screw thread lead; (7) the stack diameter; (8) the stack length; and (9) the stator diameter of the HUSM. Refer to Figure 3 and Figure 9 for the schematic of the various parameters. The objective here was to improve the actuator’s power density, a significant metric in aerospace applications. To this end, the following model treatments were required: to begin with, for convenience in the model’s modification and comparison, the generalized actuator parameter multiplication factors pertaining to the original actuator design instead of the actual numerical values were used for the mathematical model. For example, the stack length multiplication factor of 1.0 represents the original stack length, while that of 2.0 means double the original stack length. Then, the relationships between the parameter multiplication factor and the stiffness, mass, and other mechanical characteristics of each related component in the actuator were derived according to the modeling methods described in Section 3.2 and the actual experimental performance summaries, so that a parameterized actuator model reflecting these relationships could be established. Finally, the simulated actuator power and power density were computed by recording the actuator’s average speed, external load, and total mass in a feasible space of the generalized parameter multiplication factor.

**Figure 11:**
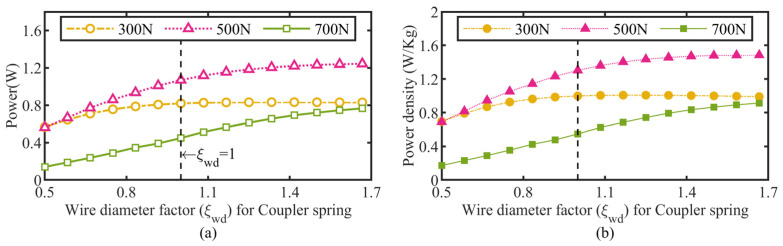
Effects of wire diameter of coupler spring on actuator performance. (**a**) Power versus wire diameter factor; (**b**) power density versus wire diameter factor.

As the most compliant part, the stiffness of the torsion spring couplers dominated by its spring wire diameter may significantly impact the prototype’s displacement-accumulated motion. Therefore, the coupler wire diameter factor *ξ*_wd_ was chosen for investigation first, and its influence on the actuator’s output power and power density were calculated using a loop program called the parameterized actuator model in MATLAB and plotted in Figure 11. The simulated actuator power versus the coupler wire diameter factor performance demonstrates a similar trend to its power density versus the coupler wire diameter factor, and the power density shows a nearly linear increase initially, then gradually tends to saturate following the incremental coupler wire diameter for all three actuation loads, which is probably because the actuator’s output benefits from the enhancement of the clamping effect as the coupler stiffness increases and is then limited by other structural parameters. Moreover, the coupler wire diameter for the optimal power density output increases with increasing actuation loads. All these behaviors imply that a larger coupler wire diameter is beneficial for the actuator’s output.

**Figure 12:**
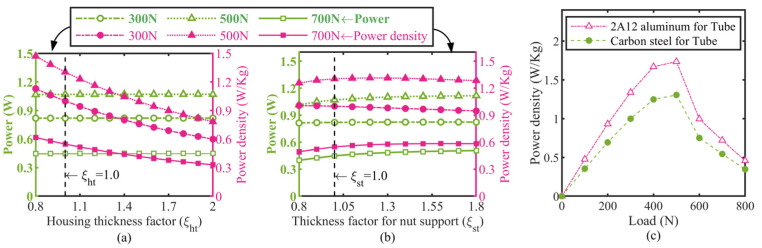
Simulated results of several critical parameters in actuator support components. Both power and power density versus: (**a**) housing thickness factor, (**b**) nut support thickness factor, (**c**) material of tube in housing.

The thickness of the housing and nut support, and the tube material properties determine their own structural compliance and mass when keeping the other parameters constant, so the values of these parameters not only considerably affect the displacement loss in the PZT stack’s stroke that is transferred to the load but also dramatically influences the actuator’s total mass due to their bulky volume. Therefore, the corresponding parameter factors of these components as arguments of the output power and power density were simulated and shown in Figure 12a,b,c, respectively. Several insights can be gained from the results. First, the predicted actuator’s power output displayed in Figure 12a does not vary nearly with increasing the housing thickness factor *ξ*_ht_ for all the applied loads, which of course gives the result that the actuator’s power density declines rapidly with the rise of *ξ*_ht_, as shown in Figure 12a. This also indicates a significant thing about the prototype: a thinner housing would bring a higher power density. Secondly, the model predicts a slight increase in the power density with the increase in the nut support thickness for actuation loads above 300 N, and the actuator’s power density peaks at approximately 1.32 W/kg against a nut support thickness multiplication factor of 1.2. Finally, the peak power density would reach a 33% increase at the concerned 500 N rated load if the tube material in the actuator’s housing was replaced by 2A12 aluminum.

**Figure 13:**
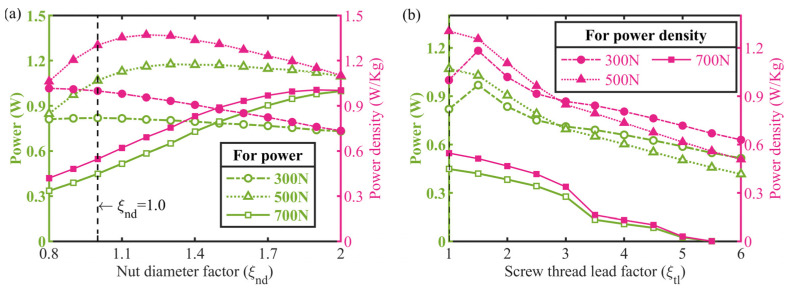
Effects of critical parameters in clamping components on actuator output. Both power and power density versus: (**a**) nut diameter factor, (**b**) screw thread lead factor.

Generally, the nominal diameter and thread lead parameters determine the stiffness, inertia, and mass of the clamping components such as the feed screw, clamping nuts, and so on, which may greatly influence the actuator’s displacement-accumulated motion. The nut diameter and thread lead factors were chosen as the independent variables for the design analysis and optimization, respectively. Figure 13 plots the predicted output power and power density versus the above parameter factor curves, respectively. A notable observation in Figure 13a is that there is an optimal clamping nut diameter, above which the peak power does not increase while the peak power density presents a roll-off trend for a 500 N external load, while for external loads of 300 N and 700 N, the dependence of the actuator’s power density on the nut diameter factor trends are contrary, which reveals that the screw–nut diameter plays an important role in the response of the system. In addition, it is evident from Figure 13b that a larger thread lead seems unhelpful to improve the actuator’s power and power density output for external loads above 300 N.

**Figure 14:**
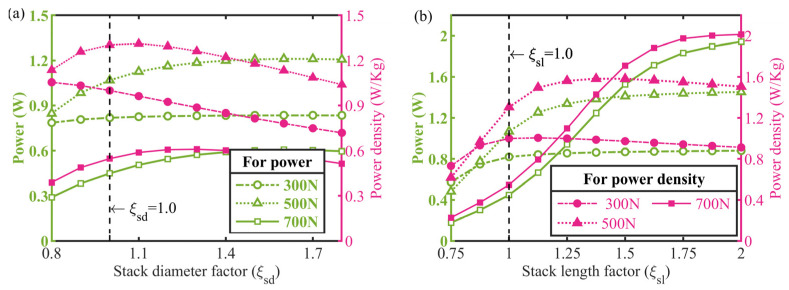
Effects of critical parameters in PZT stack on actuator behaviors. Power and power density versus: (**a**) stack diameter factor, (**b**) stack length factor.

As a core drive unit, the PZT stack’s output characteristics determine the stall load and play a critical role in the actuator’s performance output. Based on a market survey and experimental statistics, the stack blocked force and free stroke were individually proportional to its sectional area and axial length at a constant drive voltage, and the ratio of the commercial stacks’ outer diameter to the inner diameter could be taken as approximately 1.4. Accordingly, parameterized mathematical models regulated by the stack’s outer diameter and length, respectively, were constructed and computed. The simulated results were plotted in Figure 14. It is obvious in Figure 14a that there is an optimal stack diameter beyond the initial value (*ξ*_nd_ = 1.0) to force the actuator to attain a peak power density for the concerned loads of 500 N and above, and the trade-off between increasing the power density and actuator mass is clear. Likewise, the optimal stack length for the actuator’s power density output is also larger than the original dimension for all the loads studied according to Figure 14b, and more notably, the peak power density increases from 0.9 W/Kg to 2.1 W/Kg with the increasing load. Hence, an important conclusion that increasing the stack diameter and length properly brings the actuator a significant gain in power density could be drawn here.

Last but not least, the effect of the HUSMs’ performance on the actuator’s response was studied with the help of model simulation because it dominates the optimal matching frequency applied to the PZT stack and thereby determines the maximum actuator output speed indirectly, assuming the available stroke of the stack during its one drive period is constant, as described in Section 3.3. During the past few decades, a vast amount of HUSMs has been developed for various applications in the Precision Drive and Control Institute of Nanjing University of Aeronautics and Astronautics where the authors work (http://www.scnuaa.com/, accessed on 8 August 2022). Over careful analysis and summaries against the performance, physical size, and mass collected from these motors, the relationships between the HUSMs’ parameters and its output performance were found. Since the length varies by little for many motor versions, the stator diameter was selected as an independent design variable to formulate the expressions for the motor stall torque, free speed, and mass, thus giving:

(11) $$n_{f}=\left(8.26e-2D_{u}{}^{2}-0.39D_{u}+7.2\right)/\left(6.4e-11D_{u}{}^{5.556}+0.04\right)$$

(12) $$T_{b}=1.3e^{-10}D_{u}{}^{5.556}+0.08$$

(13) $$M_{u}=2.8e^{-4}D_{u}{}^{2}-0.018D+0.34$$

where *T*_b_, *n*_f_, *M*_u_, and *D*_u_ are the motor stall torque (N·m), free speed (r/min), mass (Kg), and stator diameter (mm) in sequence.

**Figure 15:**
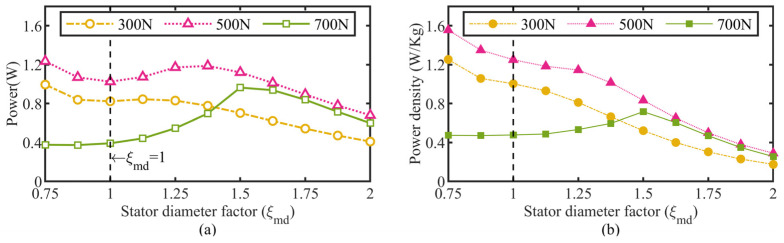
HUSM stator diameter’s effect on actuator performance. (**a**) Power and (**b**) power density versus stator diameter factor.

Inputting the above empirical relationships to the dynamic model, the actuator’s power and power density as a function of the stator diameter factor were simulated and plotted in Figure 15. The actuator’s power density experiences a more rapid decline than the output power does with an increase in the stator diameter for a load range of 300 N–500 N, but rises slowly up to a 0.8 W/Kg peak at the stator diameter factor of 1.5 for the 700 N load, which implies that a low-speed and large-torque characteristics determined by a larger HUSM stator diameter is more conducive to the actuator’s power density output at heavy loads, while the reverse case occurs for light loads. Considering the levels of the peak power density output, the actuator is suitable for driving its peak power load (rated load) of about 500 N and performs better at a smaller stator diameter, such as 75% of the original size.

## 6. Conclusions

To further improve the performance of the preliminary design concept, this paper developed a dynamic mathematical model and conducted a system of parameter studies and optimization analysis for a novel high-force piezoelectric actuator prototype, manufactured recently by the authors’ research group. On the basis of previously published quasi-static actuator modeling and optimization techniques, the model was built with full consideration of the nonlinear output characteristics of the critical drive components involving the PZT stack and HUSM, and the elaborate modeling of the complex actuator housing structure. Then it was experimentally validated for its accuracy and effectiveness. Finally, through the parameterized modeling and simulation against several significant design parameters, some advisable optimization directions for the actuator design were attained and outlined as follows: a larger coupler wire diameter, a thinner housing thickness, the aluminum alloy material instead of the old one for the housing tube, approximately 1.2 times of the original nut diameter, a factor of a 1.1 increase in the stack diameter, and a smaller HUSM diameter would contribute to a higher actuator power density output. The efforts on modeling and the optimization of the parameter not only reveal the actuator’s great potential for performance improvement, but they also provide useful references for the design of intermittent clamping type inchworm actuators.

## Data Availability

Data underlying the results presented in this paper are not publicly available at this time but may be obtained from the authors upon reasonable request.
